# The improvement effects and mechanisms of virtual reality training on depression: a systematic review from a neurotransmitter-plasticity dual-pathway perspective

**DOI:** 10.7189/jogh.16.04007

**Published:** 2026-02-27

**Authors:** Liang Sun, Lanfang Luo, Tingran Zhang, Yi Yang, Chuanqiushui Wang, Jiong Luo

**Affiliations:** 1 Physical Education Institute, Southwest University, Chongqing, China; 2 Department of Physical Education, Chongqing Mining Engineering School, Chongqing, China

## Abstract

**Background:**

Depression is a common mental disorder and a leading cause of disability, affecting approximately 280 million people worldwide. Its pathological mechanisms are closely linked to neurotransmitter homeostasis imbalance and neuroplasticity impairment. We aimed to systematically evaluate the clinical efficacy of virtual reality (VR) training in improving depression and to elucidate its dual-pathway regulatory mechanism involving neurotransmitter-plasticity interactions.

**Methods:**

We systematically searched PubMed, Web of Science, CNKI, and ScienceDirect databases from their formation to May 2025 to identify randomised controlled trials (RCTs) assessing VR-based interventions. Given the significant heterogeneity across protocols, we conducted a narrative synthesis in accordance with the Synthesis Without Meta-analysis (SWiM) reporting guidelines rather than a quantitative meta-analysis. We assessed the quality of the included studies using the Physiotherapy Evidence Database (PEDro) scale.

**Results:**

Based on 16 studies, we found that VR training can significantly alleviate depressive symptoms through multimodal sensory stimulation and immersive interactions, as the included RCTs generally reported reductions in depression scale scores. Preliminary evidence from several studies also suggests a positive correlation between clinical efficacy and biomarkers of neural structure recovery and functional network synchronisation. However, due to high heterogeneity in intervention protocols, control group designs, and outcome measures, we were unable to provide a unified estimate of the VR training efficacy.

**Conclusions:**

Evidence suggests that VR training may reverse the pathological cycle of depression through a synergistic dual pathway of neurotransmitter regulation and neuroplasticity enhancement, offering a novel strategy for precision intervention. Future research should optimise VR intervention protocols, explore synergistic effects with traditional therapies, and validate its long-term efficacy and safety in special populations.

**Registration:**

PROSPERO: CRD420261300373.

Depression is a common mental disorder and one of the most disabling worldwide, affecting around 3.8% of the global population, or approximately 280 million people [1]. It is closely associated with an imbalance in neurotransmitter homeostasis and impaired neuroplasticity [2]. While the classic monoamine hypothesis laid the theoretical foundation for the development of antidepressant drugs, the suboptimal response of some patients to pharmacotherapy suggests that the pathological mechanisms of depression may involve more complex molecular network regulation and dynamic neuroplasticity imbalances [3,4]. Research indicates that dysregulation of neurotransmitters, such as serotonin (5-HT), glutamate, and γ-aminobutyric acid (GABA), not only directly disrupts synaptic transmission but also exacerbates neuroplasticity damage by suppressing the secretion of brain-derived neurotrophic factor (BDNF). This forms a pathological cycle of neurotransmitter imbalance-neuroplasticity impairment, ultimately leading to structural degeneration and functional network abnormalities in key brain regions such as the hippocampus and prefrontal cortex [5,6].

These multidimensional pathological features provide a critical theoretical basis for exploring novel non-pharmacological interventions. Virtual reality (VR) technology provides multimodal sensory stimulation and immersive environmental interaction, showing unique therapeutic potential in neuromodulation [7,8]. Preliminary studies suggest that VR training may achieve multi-target synergistic effects through a dual regulatory mechanism: at the molecular level, it may promote the biosynthesis of monoamines and balance the function of the glutamate/GABA system, while at the synaptic level, it may enhance dendritic spine remodelling and long-term potentiation (LTP) effects [5].

However, before its application in clinical practice, the efficacy of VR technology must be evaluated and its mechanisms more deeply understood. With this in mind, we wanted to systematically evaluate the efficacy of VR training in improving depressive symptoms based on existing randomised controlled trials (RCTs). For our secondary objective, we sought to explore the potential neurotransmitter-plasticity dual-pathway mechanism by which VR training affects depression to provide a theoretical basis for optimising intervention protocols and future research.

## METHODS

We registered the protocol for this systematic review in PROSPERO (CRD420261300373). We conducted and reported this review in accordance with the PRISMA 2020 statement [9].

### Literature search strategy

We systematically searched PubMed, Web of Science, CNKI, and ScienceDirect from their formation to May 2025. We systematically searched PubMed, Web of Science, CNKI, and ScienceDirect from their formation to May 2025. The search strategy (**Online Supplementary Document**) was constructed based on three key concepts: virtual reality technology, depression or depressive disorders, and neurobiological mechanisms (*e.g.* neurotransmitters, neuroplasticity, or BDNF). We also reviewed the reference lists of included studies to ensure comprehensiveness.

### Eligibility criteria

We included RCTs and crossover RCTs reported in English or Chinese, provided they fit the following criteria:

− Population: patients diagnosed with depression or study populations where depressive symptoms were a primary outcome measure. We did not apply any restrictions on age, gender, or health status (*e.g.* clinical *vs.* non-clinical populations) to capture a broad evidence base and explore the heterogeneity from these factors in the subsequent analysis.
− Intervention: experimental group received VR-based intervention, such as VR exercise, games, or psychotherapy using head-mounted displays (HMDs) or screen-based immersion.
− Control: control group did not receive VR-based intervention, but only usual care or an alternative non-VR intervention (*e.g.* traditional exercise).
− Outcomes: included at least one standardised depression symptom assessment scale (*e.g.* Beck Depression Inventory (BDI), Hamilton Depression Rating Scale (HAM-D), Geriatric Depression Scale (GDS), or biomarkers related to neurotransmitters or neuroplasticity (*e.g.* BDNF, cortisol).

We excluded reviews, case reports, conference abstracts, non-experimental studies (*e.g.* observational studies), studies unrelated to VR technology, and any duplicate publications.

### Study selection and data extraction

Two researchers (TZ, YL) independently screened the titles and abstracts of the retrieved records, after which they reviewed the full texts of any remaining literature to determine final inclusion. A third researcher (LS) resolved any disagreements during the screening process. Then, two researchers (YY, CW) independently extracted the following data using a pre-designed extraction form (Table S1 in the **Online Supplementary Document**): study information (author, year), study design, participant characteristics (sample size, age, gender), detailed information on intervention and control measures (type, duration, frequency, assessed according to the TIDieR checklist), outcome measures, and main results (including effect sizes, confidence intervals, or *P*-values) [10].

### Bias and quality assessment

We used the Physiotherapy Evidence Database (PEDro) scale [11]. to assess the methodological quality of the included RCTs. The scale consists of 11 items, with a higher total score indicating better methodological quality. Given the nature of VR interventions, therapist blinding is often difficult to achieve, meaning that studies rarely met this criterion. However, we maintained the standard scoring (10 points) and did not remove this item from the assessment. We defined studies with a score ≥5 as high-quality. Two researchers (LS, TZ) independently performed the assessment and discussed the final consensus to ensure consistency. To inform clinical practice, we assessed the strength of the evidence using the GRADE-CERQual framework.

### Data synthesis

Given the significant clinical and methodological heterogeneity among the included studies in terms of VR technology, intervention protocols, control conditions, and outcome measurement tools, we did not perform a quantitative synthesis (meta-analysis) to estimate a unified effect size. Instead, we employed a narrative synthesis approach that followed the SWiM guideline.

## RESULTS

**Figure 1:**
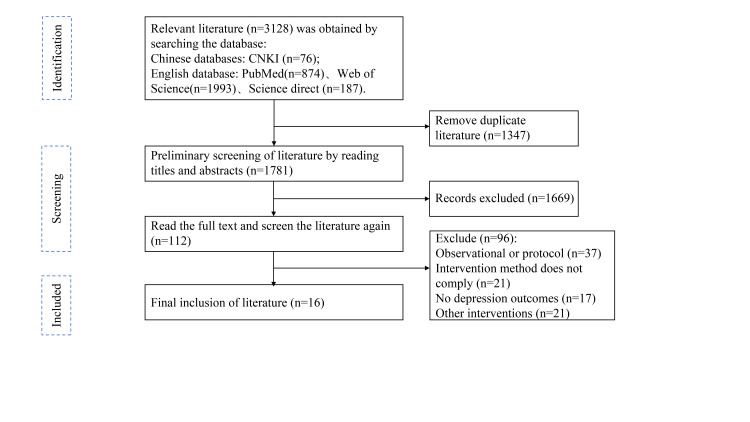
Flowchart regarding the selection process of the scientific studies.

We retrieved 3128 articles in the initial search and removed 1347 duplicates, leaving 1835 studies for title and abstract screening. We then removed 1732 irrelevant records and screened the full texts of the remaining 112 studies. Ultimately, we selected 16 RCTs that met the inclusion criteria (**Figure 1**). They were published between 2017 and 2025 and involved 1998 participants [12–27]. The study populations were heterogeneous and included women with postpartum depression, post-stroke patients, older individuals, and general patients with depression. The VR interventions were also diverse and included VR cycling, VR-enhanced mindfulness and yoga, VR horticultural activities, and VR therapy combined with transcranial magnetic stimulation. The duration, frequency, and total period of interventions varied significantly across studies (**Table 1**). All of the studies scored ≥5 on the PEDro scale and were classified as high quality. However, despite their overall quality, our assessment showed that studies commonly failed to meet the criteria for blinding (particularly for therapists and participants) due to the inherent difficulty of concealing VR interventions

**Table 1:** VR training studies on depression improvement

		Study participants			Intervention details					
**Author, year, reference**	**Country**	**Age in years, VR; CG**	**n**	**Gender, male/female**	**Type**	**Duration, in minutes**	**Frequency, sessions/week**	**Period**	**Key quantitative results**	**Conclusion**
Turoń-Skrzypińska *et al.*, 2023, [12]	Poland	63; 64	39; 46	58/27	Cycling	20	3	3 months	Beck (change) x̄ = −1.19 (SD) = 1.97/x̄ = 2.56 (SD) = 5.07); GAD7 (change) x̄ = −1.84 (SD) = 2.30/x̄ = 1.48 (SD) = 1.48)	Significant reduction in depressive symptoms
Veling *et al.*, 2021, [13]	Netherlands	41.6	50	17/33	VRelax	10	7	10 days	Total negative affective state (change: 21.2% *vs.* 16.2%; t_1684_ = −2.02, 95% CI: −18.70, −0.28; *P* = 0.04)	Significant reduction in negative affect
Fan *et al.*, 2022, [14]	China	x̄ = 70.94 (SD = 5.0); x̄ = 69.8 (SD = 3.8)	32; 30	11/51	Horticultural activity	120	1	8 weeks	GEE self-esteem (*β* = 2.18; *P* = 0.005) and mastery (*β* = 1.23; *P* = 0.039)	Improved self-esteem and reduced depression in elderly
Rutkowski *et al.*, 2022, [15]	Poland	x̄ = 57.8 (SD = 4.9)	32	20/12	Cycling		5	3 weeks	HADS (VR: 6.9 (3.9) *vs.* 4.7 (3.5); *P* = 0.008; CG:7.64 (4.5) vs 6.6 (4.8); *P* = 0.017)	Reduced anxiety and depressive symptoms
Liu *et al.*, 2025, [16]	China	x̄ = 30.5 (SD = 5.3); x̄ = 30.5 (SD = 5.4)	29; 28	0/81	VR-enhanced mindfulness and yoga	60	3	8 weeks	EPDS: *P* < 0.001, ηp2 = 0.18; GAD-7: *P* < 0.001, ηp2 = 0.17	Reduced postpartum depression and anxiety post-COVID-19
Qiu *et al.*, 2024, [17]	China	62	212	0/212	Tai chi		1	6 months	GDS: *P* ≤ 0.05	Enhanced efficacy of Tai Chi combined with VR in elderly
Kiper *et al.*, 2022, [18]	Poland	x̄ = 65.5 (SD = 6.7); x̄ = 65.6 (SD = 5.0)	30; 30	0/60	Neurorehabilitation training	60		6 weeks	GDS: ηp2 = 0.13; *P* < 0.01	Improved mood and reduced depression in post-stroke patients
Smilovich *et al.*, 2023, [6]	Russia	x̄ = 42.08 (SD = 18.2)	85	15/70	Transcranial magnetic stimulation		5	20 days	HAM-D: 76% *vs.* 37%, (*P* = 0.0033)/43% (*P* = 0.0047)	Effective and safe for depression treatment
Jimenez-Barragan *et al.*, 2025, [20]	Spain	x̄ = 31.9 (SD = 4.8)	70	0/70	Mindfulness	14	7	6 weeks	EPDS: decrease from 11.32 (SD = 0.96) to 7.25 (SD = 1.32; *P* < 0.001)	Significantly reduced anxiety and depression
Vieira *et al.*, 2018, [21]	China	x̄ = 42.1 (SD = 7.3); x̄ = 42.5 (SD = 5.5)	33; 31	13/51	Integrated exercise	60	3	6 months	DASS-21: *P* = 0.001	No significant improvement in anxiety/depression
Blázquez-González *et al*., 2024, [22]	Spain	x̄ = 54.2 (SD = 6.6); x̄ = 54.3 (SD = 7.2)	17; 41	37/21	Switch games	20	1	6 weeks	HADS: *P* = 0.01	Beneficial effects on neurological and anxiety states in stroke patients
Seo *et al*., 2023, [23]	South Korea	x̄ = 47.4 (SD = 5.5); x̄ = 48.3 (SD = 7.6)	23; 24	0/70	Cycling	50	3–5	8 weeks	PHQ-9: F = 3.462: *P* < 0.001	Improved depression levels and exercise immersion in overweight women
Turrado *et al.*, 2021, [24]	USA	64; 68	58; 68	80/46	Surgery simulation				HADS: 4.00 (3.00; 7.00) *vs.* 5.00 (3.00–7.00); *P* < 0.247	Reduced perioperative anxiety in colorectal cancer patients
Beidel *et al.*, 2017, [25]	USA	x̄ = 37.7 (SD = 8.5); x̄ = 33.3 (SD = 11.3)	49; 43	86/6	Virtual battlefield	60	1–3	17 weeks	HAM-D: (*β* = −41.73, SE = 3.94, *t* = −10.59; *P* < 0.001, R2 = 0.383)	Significant reduction in depressive symptoms
Huang *et al.*, 2022, [26]	China	x̄ = 58.3 (SD = 11.2); x̄ = 50.8 (SD = 12.3)	15; 15	10/20	VR motion games	60	2–3	16 sessions	BDNF: +4.87%; *P* = 0.023	Mild improvement in BDNF expression
Connelly *et al.*, 2024, [27]	Australia	x̄ = 23.0 (SD = 4.53)	20	7/13	Story-based activity	12	1	7–19 days	MEP amplitude: r = −0.72; *P* < 0.001	Enhanced neural plasticity

BDNF – brain-derived neurotrophic factor, CG – control group, CI – confidence interval, DASS-21 – Depression Anxiety and Stress Scale 21, EPDS – Edinburgh Postnatal Depression Scale, GAD-7 – Generalised Anxiety Disorder-7, GEE – Generalised Estimating Equation, GDS – Geriatric Depression Scale, HADS – Hospital Anxiety and Depression Scale, MEP –motor evoked potential, PHQ-9 – Patient Health Questionnaire-9, SD – standard deviation, VR – virtual reality, x̄ – mean

### Overall effect of VR training on depressive symptoms

Most of the included studies reported that VR training significantly improved depressive symptoms. For example, Turoń-Skrzypińska and colleagues found that VR cycling significantly reduced BDI scores in haemodialysis patients [12], and Liu and colleagues found that VR-enhanced mindfulness and yoga effectively reduced Edinburgh Postnatal Depression Scale scores in postpartum women [16]. Moreover, Beidel and colleagues showed significantly reduced HAM-D scores in veterans through virtual battlefield exposure therapy [25]. Only one study by Viera and colleagues reported that a VR-based integrated exercise programme in cardiac rehabilitation did not show a significant improvement in anxiety or depression on the Depression Anxiety and Stress Scale 21 scale [21].

### Effects of VR training on neurobiological markers

While measuring neurobiological markers, Huang and colleagues found that VR-based motor games led to a mild improvement in BDNF expression in stroke patients [26]. Connelly and colleagues found that, by measuring motor evoked potential amplitude, VR activity could enhance neuroplasticity [27]. These findings provide preliminary evidence for VR’s impact on neuroplasticity, but the number of such studies is limited, and more high-quality evidence is needed to validate these effects.

## DISCUSSION

We integrated findings from 16 RCTs to evaluate the efficacy of VR training for depression and to explore its potential neurotransmitter-plasticity dual-pathway mechanism. Our results suggest that, despite significant methodological heterogeneity, VR as a non-pharmacological intervention could be useful in improving depressive symptoms.

### A comprehensive interpretation of the neurotransmitter-plasticity dual-pathway hypothesis

**Figure 2:**
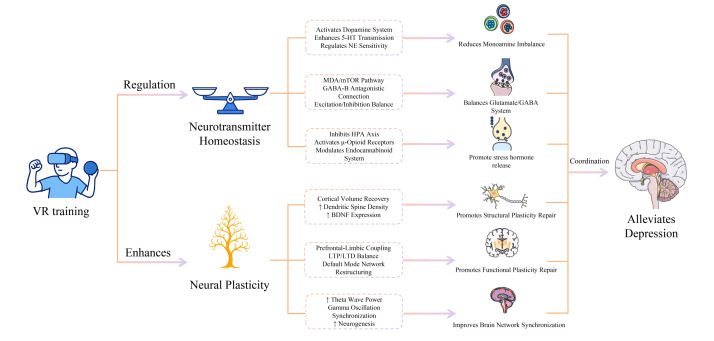
Mechanism of VR training improving depression by regulating neurotransmitter homeostasis and enhancing neural plasticity. BDNF – brain-derived neurotrophic factor, GABA – gamma-aminobutyric acid, HPA axis – hypothalamic-pituitary-adrenal axis, LTD – long-term depression, LTP – long-term potentiation, mTOR – mechanistic target of rapamycin, NE – norepinephrine, NMDA – N-methyl-D-aspartate, 5-HT – 5-hydroxytryptamine (serotonin).

Our core findings support a hypothetical model (**Figure 2**) where VR training exerts its antidepressant effects synergistically through two interconnected pathways: by regulating neurotransmitter homeostasis and by enhancing neuroplasticity. At the neurotransmitter level, immersive and rewarding environments created by VR may improve core symptoms (*e.g.* anhedonia) by activating the mesolimbic dopamine system [28]. This regulation goes beyond the single-target approach of traditional drugs and may simultaneously influence the 5-HT and norepinephrine systems [29,30], as well as modulate the glutamate/GABA balance [31]. Moreover, by providing engaging experiences, VR may suppress the hyperactivity of the HPA axis [32], reduce the secretion of stress hormones like cortisol [33], and activate endogenous analgesic systems (*e.g. β*-endorphins and endocannabinoids) [34,35]. However, these mechanistic claims are mostly derived from animal models or indirect biomarker measurements (*e.g.* peripheral blood tests), and direct evidence from the human brain remains scarce [36]. Caution is, therefore, necessary when translating findings from animal studies to human clinical contexts. The evidence appears more robust at the neuroplasticity level; VR training, especially when combined with exercise, is thought to promote neurogenesis in the hippocampus, increase dendritic spine density, and repair functional connectivity in the prefrontal-limbic system [5,37,38]. The dose-dependent relationship observed in clinical studies between the improvement of depressive symptoms and the increase in hippocampal volume or prefrontal cortical thickness supports this pathway [7]. VR may enhance brain network plasticity and information processing efficiency by inducing LTP and modulating neural oscillations such as θ-η waves [39,40].

### Methodological heterogeneity and key confounding factors

The significant methodological heterogeneity across the included studies prevented us from drawing definitive conclusions. The development of VR technology is a key issue in this sense, as we were unable to combine studies of early low-fidelity VR systems with those using modern, highly immersive head-mounted displays (HMDs). Future studies should clearly report the technical parameters of the VR systems used and consider stratified analysis based on technology levels. The ambiguity of the content of interventions within the included RCTs is another challenge, as we encountered vague descriptions of interventions, such as ‛rigorously designed exercise protocols’ [41]. Furthermore, many studies combined VR with physical exercise (*e.g.* cycling, tai chi) [17,23]. This raises a core confounding issue: how much of the observed efficacy is attributable to the immersive experience of VR, and how much comes from the known antidepressant effects of physical exercise itself (*e.g.* promoting BDNF secretion) [42,43]? Future studies must adopt more rigorous control designs, for example, by including a ‛VR without exercise’ group and an ‛exercise without VR’ group, to disentangle these confounding variables. We strongly recommend that future trials follow the TIDiER checklist to detail every component of the intervention. Finally, the included studies varied greatly in population characteristics, intervention duration, frequency, and outcome measures, preventing us from performing a statistical meta-analysis.

Some literature suggests that combining VR with brain-computer interfaces can achieve closed-loop regulation of neural activity [8,44]. However, we must recognise that such technology is still in its preliminary exploratory stage, mainly limited to a few pilot studies. Its technical complexity, high cost, and reliance on specialised operators are major barriers to its widespread clinical application. When discussing its potential, one should remain cautious and frankly acknowledge the current practical challenges.

### Limitations

This study has several limitations. First, our search strategy did not include grey literature (e.g., dissertations, conference proceedings), which might have led to us overlooking some relevant studies and, consequently, to publication bias. Second, we limited the search to studies reported in English and Chinese, which could lead to language bias. Third, due to the high heterogeneity of the included studies, we did not perform a meta-analysis. Consequently, a quantitative assessment of publication bias (e.g., funnel plots) was not conducted. Finally, although we assessed the quality of the studies, most included trials failed to meet the blinding criteria (particularly for therapists and participants) due to the inherent difficulty of concealing VR interventions, which could affect the reliability of the results.

### Implications for clinical practice and future research

Despite these limitations, we found important insights for clinicians and policymakers. VR training, as a safe, controllable, and engaging intervention, is particularly suitable for patients (*e.g.* postpartum, elderly, and post-stroke depression populations) who have a poor response to or cannot tolerate traditional treatments [16–18]. The most significant practice is the standardisation of intervention protocols and the validation of biomarkers, which we also recommend for future research.

## CONCLUSIONS

Our findings suggest that VR training, as an innovative non-pharmacological intervention, could help alleviate symptoms of depression. Its mechanism of action may involve the synergistic effect of a neurotransmitter-plasticity dual pathway. At the functional level, VR training may restore neurotransmitter homeostasis by regulating monoamine, glutamate/GABA systems, and the HPA axis [5,34], and at the structural level, it may enhance neuroplasticity by promoting hippocampal neurogenesis and repairing connectivity in key brain regions [37,45]. However, the current evidence base is limited by study heterogeneity, potential confounding factors, and an insufficient understanding of the mechanisms behind the observed effects. Future research should focus on developing standardised intervention protocols, conducting more rigorous RCTs, and more deeply exploring the underlying neurobiological mechanisms [7,8].

## Additional material


Online Supplementary Document
