# Supplementary material for: The improvement effects and mechanisms of virtual reality training on depression: a systematic review from a neurotransmitter-plasticity dual-pathway perspective
Source: J Glob Health. 2026 Feb 27;16:04007. doi: 10.7189/jogh.16.04007 (PMC12947600; doi:10.7189/jogh.16.04007)
Supplement: Online Supplementary Document [file jogh-16-04007-s001.pdf]

# Supplement to: Sun L, Luo L, Zhang T, Yang Y, Wang C, Luo J. The improvement effects and mechanisms of virtual reality training on depression: a systematic review from a neurotransmitter-plasticity dual-pathway perspective. J Glob Health. 2026;16:04007.

## Supplementary material

## 1 - Literature search strategy

The search keyword combinations included (“virtual reality” OR “VR”) AND (“depression” OR “depressive disorder”) AND (“neurotransmitter” OR “neuroplasticity” OR “BDNF”). To ensure comprehensiveness, we also reviewed the reference lists of included studies. We used Boolean operators “AND” and “OR” to combine keywords; for example, a typical search strategy in PubMed was: (((“Virtual Reality”[Mesh]) OR “Virtual Reality Exposure Therapy”[Mesh] OR “VR” OR “Immersive” OR “Exergam*”)) AND ((“Depression”[Mesh]) OR “Depressive Disorder, Major”[Mesh] OR “Depress*”)) AND ((“Neurotransmitter Agents”[Mesh] OR “Neurotransmitter*”) OR (“Neuronal Plasticity”[Mesh] OR “Neuroplasticity” OR “BDNF”))

## Table S1 - Data Extraction Proforma

| Extraction Item | Description/Content to Fill |
|---|---|
| **Basic Information** | |
| Country | |
| Study Design | |
| **Participant Characteristics** | |
| N, T/C | |
| Age, Mean±SD or Range | |
| Gender, M/F | |
| Diagnostic Criteria/Population | |
| **Intervention Details - TIDIER Checklist** | |
| Intervention Group | |
| Control Group | |
| Duration per session | |
| Frequency | |
| Total Period | |
| **Outcome Measures** | |
| Primary Outcomes | |
| Secondary Outcomes | |
| **Key Findings** | |
| Within-group change | |
| Between-group difference | |

| **Quality Assessment** |
|---|
| PEDro Score |
| Notes |

# Table S2 - Physiotherapy Evidence Database score of the included studies

| Author, year | Eligibility criteria specified | Randomization | Concealed allocation | Baseline comparability | Patients blinded | Care provider blinded | Adequate follow up | Intention-to-treat | Between-group comparisons | Score |
|---|---|---|---|---|---|---|---|---|---|---|
| Turoń-Skrzypińska A, 2023[9] | + | + | - | + | - - - | + | + | + | + | 6 |
| Veling W, 2021[10] | + | + | - | + | - - - | + | + | + | + | 6 |
| Fan CC，2022[11] | + | + | - | + | - - - | + | + | + | + | 6 |
| Rutkowski S , 2022[12] | + | + | + | + | - - - | + | + | + | + | 6 |
| Liu N, 2025[13] | + | + | + | + | - - - | + | + | + | + | 6 |
| Qiu T，2024[14] | + | + | + | + | - - - | + | + | + | + | 6 |
| Kiper P，2022[15] | + | + | + | + | - - - | + | + | + | + | 6 |
| Smilovich AA，2023[6] | + | + | + | + | - - + | + | + | + | + | 7 |
| Jimenez-Barragan M，2025[17] | + | + | - | + | - - - | + | + | + | + | 6 |
| Vieira Á，2018[18] | + | + | - | + | - - - | + | + | + | + | 6 |
| Blázquez-González P, 2024[19] | + | + | - | + | - - - | + | + | + | + | 6 |
| Seo EY, 2023[20] | + | + | - | + | - + - | + | + | + | + | 7 |
| Turrado V, 2021[21] | + | + | + | + | - - - | + | + | + | + | 6 |
| Beidel DC, 2017[22] | + | + | - | + | - - - | + | + | + | + | 6 |
| Huang CY, 2022[23] | + | + | - | + | - - - | + | + | + | + | 6 |
| Connelly N, | + | + | - | + | - - - | + | + | + | + | 6 |

2024[24]

## Table S3 -GRADE-CERQual Evidence Quality Assessment

| Finding | Summary of Evidence | Methodological Limitations | Coherence | Adequacy of Data | Relevance | CERQual Assessment of Confidence |
|---|---|---|---|---|---|---|
| 1. VR training improves depressive symptoms | In the 16 included RCTs, 15 reported that VR training significantly improved depressive symptoms compared to control groups. These studies covered various VR intervention types, populations, and depression assessment scales. | Moderate concerns: Although all studies were high-quality RCTs, most faced common challenges in achieving blinding of therapists and participants, which could introduce performance bias. | Minor concerns: The direction of the effect is highly consistent (15/16 studies reported a positive effect). However, due to the diversity of intervention protocols and populations, the magnitude of the effect size likely varies. | Moderate concerns: Although the number of studies is sufficient, there is significant heterogeneity in intervention protocols, control group setups, and outcome measures, which prevented a meta-analysis to obtain a unified effect size estimate. | No or minor concerns: The interventions, populations, and outcomes of all included studies are directly relevant to the core review question. | Moderate |
| 2. Effect of VR training on neurobiological markers (e.g., BDNF) | Only one included study measured BDNF levels, reporting a 'mild improvement' in BDNF expression in patients with chronic stroke | Serious concerns: The evidence relies entirely on a single study, making it impossible to assess the risk of bias across studies. | Not applicable: As there is only one data point, coherence cannot be assessed. | Serious concerns: The data is extremely sparse, derived from only one study with a relatively small sample size. The | No or minor concerns: This outcome measure is highly relevant to the mechanistic exploration of the | Low |

| | | | | | | |
|---|---|---|---|---|---|---|
| | following a VR-based motor game intervention. | | | manuscript explicitly states that more high-quality evidence is needed to confirm this finding. | review. | |
| 3. Effect of VR training on neuroplasticity (e.g., MEP) | Similarly, only one included study assessed neuroplasticity by measuring motor evoked potential (MEP) amplitude, reporting that VR activity could 'enhance neuroplasticity' . | Serious concerns: The evidence relies entirely on a single study, which was described as a preliminary 'pilot trial'. | Not applicable: As there is only one data point, coherence cannot be assessed. | Serious concerns: The data is extremely sparse, originating from a single pilot study. The manuscript clearly states more evidence is needed for confirmation. | No or minor concerns: This outcome measure is highly relevant to the mechanistic exploration of the review. | Low |

## Table S4 -Summary of Methodological Heterogeneity Across Included Studies

| Study ID | VR Technology | Control Condition | Frequency & Period | Population | Combined with Exercise? |
|---|---|---|---|---|---|
| Turoń-Skrzypińska, 2023 | NefroVR | No intervention | 3 sessions/week , for 3 months | Hemodialysis patients | Yes |
| Veling, 2021 | VRelax relaxation app | Cross intervention | 7 sessions/week , for 10 days | Inpatients with psychiatric disorders | No |
| Fan CC, 2022 | 3D VR Horticultural Activity | —— | 1 session/week, for 8 weeks | Community-dwelling elderly | No |
| Rutkowski, 2022 | VR TierOne (head-mounted display) | Traditional forms of training | 5 sessions/week , for 3 weeks | Post-COVID-19 patients | Yes |
| Liu N, 2025 | Oculus Quest 2 | No | 3 | Postpartum women | Yes |

| | | | | | |
|---|---|---|---|---|---|
| | VR | intervention | sessions/week, for 8 weeks | | |
| Qiu T, 2024 | Oculus Quest 2 headset and hand-tracking technology | Traditional forms of training/ No intervention | 1 session/week, for 6 months | Elderly | Yes |
| Kiper P, 2022 | VR TierOne (consists of original software integrated with HTC VIVE PRO Goggles ) | Traditional forms of training | [NR], for 6 weeks | Post-stroke patients | Yes |
| Smilovich, 2023 | —— | intermittent theta flashes | 5 sessions/week, for 20 days | Patients with adynamic depression | No |
| Jimenez-Barragan, 2025 | Oculus Go (Meta Reality Labs) virtual reality headset | standard care | 7 sessions/week, for 6 weeks | Pregnant women | No |
| Vieira Á, 2018 | Kinect-RehabPlay system( composed of three modules, the virtual reality environment, the Kinect sensor, and the monitoring software package) | Usual Care | 3 sessions/week, for 6 months | Cardiac rehabilitation patients | Yes |
| Blázquez-González, 2024 | Nintendo Switch device and the video game Mario Party | Usual Care | 1 session/week, for 6 weeks | Stroke patients | No |
| Seo EY, 2023 | VRFit | Traditional forms of training | 3-5 sessions/week, for 8 weeks | Overweight middle-aged women | Yes |
| Turrado V, 2021 | virtual reality glasse | Usual Care | —— | Colorectal cancer surgery patients | No |
| Beidel DC, 2019 | head-mounted display (with a Sony HMZ-T3W head mounted display | Psychoeducation | 1-3 sessions/week, for 17 weeks | Patients with combat-related PTSD | No |

| | | | | | |
|---|---|---|---|---|---|
| | (HMD) | | | | |
| Huang CY, 2022 | HTC VIVE VR headset | Traditional forms of training | 2-3 sessions/week , for 16 sessions | Patients with chronic stroke | Yes |
| Connelly N, 2024 | Oculus Quest 2 headset | Transcranial Magnetic Stimulation | 1 session/week, for 7-19 days | Healthy adults | No |
